# Can visual language convey tactile experience? A study of the tactile compensation effect of visual language for online products

**DOI:** 10.3389/fpsyg.2022.1034872

**Published:** 2022-12-19

**Authors:** Xionghui Leng, Xiaoyu Zhou, Shuting Wang, Yibin Xiang

**Affiliations:** ^1^School of Economics and Management, East China Jiaotong University, Nanchang, Jiangxi, China; ^2^Jiangxi Academy of Social Sciences, Nanchang, Jiangxi, China

**Keywords:** visual language, tactile compensation, visual metaphor, online product display, mental simulation, haptic cues

## Abstract

**Introduction:**

There is a common phenomenon of tactile missing in online retail. How to realize consumer tactile compensation is a consensus problem in the field of e-commerce. More and more marketeers and scholars convey their ideas via visual display, but few researches have focused on the tactile compensatory effect of visual language.

**Methods:**

Study 1 collected data from nearly 13,000 online purchases to analyze the impact of haptic cues on sales in real online shopping platforms; Study 2 used a experimental research method to design three experimental groups: hand haptic cue group vs. Object haptic cue group vs. control group (*N* = 165) to investigate whether the main effect of haptic cues and the dual mediating effect of mental simulation held. Study 3 also adopted a simulated experimental research approach to design a two-factor group: 2 (haptic cue: hand vs. object) × 2 (product type: tactile functional product vs. tactile experiential product) (*N* = 198). To further explore whether the moderating effect of product type holds based on Study 2.

**Results:**

Therefore, based on the visualization theory and mental simulation theory, and through a second-hand data experiment and two simulated experiments, this study confirmed that visual language did have a compensation effect on tactile missing specifically. Haptic cues in metaphorical visual language can actively compensate for consumers’ tactile loss, thus affecting the purchase intention. Mental simulation plays a mediating role in the tactile compensation effect. Product type has a moderating effect, and the use of hand (object) haptic cues in metaphorical visual language in tactile functional products (tactile experiential products) can lead to a more active purchase intention.

**Discussion:**

This study not only enriches the theoretical research on the tactile compensation effect of visual language, but also provides valuable management enlightenment for e-commerce enterprises to improve the effectiveness of online product display and online sensory marketing strategies.

## Introduction

The competition in the online retail market gets more intensified due to the global COVID-19 epidemic (Yi et al., 2022). New technologies represented by the Internet, new media, cloud technology, and big data have become new driving forces for the development of media companies (Waheed et al., 2021; Yi et al., 2021), and non-contact consumption will gradually become the main consumption pattern in the post-epidemic era. Non-contact consumption makes consumers unable to truly touch products online before purchase, thus, lacking sensory experience (Yoo and Kim, 2014) and triggering great uncertainty about their purchase intention and behavior (Rathee and Rajain, 2019). Therefore, how to remedy the negative consequences caused by the naturally existing sensory barriers of e-commerce is a recognized key issue in academics. Online retailers should create an appropriate online shopping environment and develop effective visual language marketing strategies (Zheng et al., 2019) to give consumers a perfect connection between online and offline shopping experiences (Liao and Yang, 2020; Frasquet et al., 2021; Jai et al., 2021).

Online retail products primarily display *via* visual language. In this context, online consumers are unable to touch the products physically, thus, leading to heavy dependence on online sensory experience (Herz and Diamantopoulos, 2017). From the perspective of the visual language, the existing studies suggest designing visual elements containing rich tactile information to offer sensory compensation to consumers (McCabe and Nowlis, 2003; Yoo and Kim, 2014; Rodrigues et al., 2017), such as appropriate image sizes (Cornil and Chandon, 2016), vivid images to induce tactile imagery (Park, 2006), and product images with tactile information descriptions (Silva et al., 2021), to induce virtual tactile perception for tactile compensation *via* the visual language of images. However, the visual language display mentioned in existing studies is limited to the product itself, and few studies pay attention to other visual elements outside the product, such as the positive effects of haptic cues’ tactile compensation (Chen et al., 2019; Maille et al., 2020). Most of the existing research focuses on the tactile properties of products and product packaging and does not consider how haptic cues in visual language can encourage people to understand tactile properties in a metaphorical way, so as to achieve the purpose of tactile compensation. Additionally, the majority of previous investigations (Luangrath et al., 2022) have concentrated on the compensating effects of a single type of haptic cue and have not examined the possibility that tactile compensation might be achieved with various types of haptic cues. The innovative point of this paper is to delve into how online retailers can effectively design visual language, i.e., to explore designing strategies for various haptic cues types in online product displays. Through haptic cues in metaphorical visual language (i.e., MVL), the metaphorical presentation promotes consumers’ awareness of product haptic attributes and explores the positive effects, psychological mechanisms, and implementation strategies for conveying online product haptic attributes and experiences to consumers, which can be applied to the study of online consumer behavior and psychology. This paper can not only promote the research on the tactile compensation strategy of online products but also provide valuable guidance for e-commerce enterprises to implement online sensory marketing strategy.

## Literature review and hypotheses development

### Theoretical basis

#### Touch visualization theory

Neurophysiological studies have found that both tactile and visual modes share a common neural basis in the representation of external stimuli (Sathian, 2016). Further research in cognitive psychology suggests that the information observed through vision can simulate the sense of touch. This is a phenomenon known as the visual-tactile mirror mechanism (Keysers et al., 2004; Katsuyama et al., 2018; Zazio et al., 2019). Accordingly, touch visualization theory suggests that observing another person being touched, seeing an object being touched, or even any visual touch (such as a tree branch beating against a window glass) can lead to the activation of the tactile nervous system involved in the individual somatosensation (Ebisch et al., 2008; Tholen et al., 2020). The touch-sharing mechanism will be activated when watching another hand or an object being touched (Kuehn et al., 2018), meaning that participants actually perceived the haptic stimulus of their hands being touched (Kimura and Katayama, 2018; Zazio et al., 2019). This mechanism also further creates a “tactile empathy effect” (Ward et al., 2018). Thus, vicarious touch experience caused by visual touch can help individuals get physical feelings, emotional states, and emotional attitudes just like in actual touch (Ebisch et al., 2008; Fahey et al., 2019; Zeugin et al., 2020).

#### Mental simulation theory

Mental simulation refers to the imaginary representation of some events and the function or process of a series of events. It is the simulated reappearance of the event in consumers’ minds or the vicarious experiences of the described event (Taylor and Schneider, 1989). Mental simulation can be conceptualized as an automatic and unconscious form of mental imagery involving the cognitive construction of virtual situations and reconstruction of real situations and associating with the activation of brain regions involved in processing real perceptual information (Elder and Krishna, 2012). Its main function is to improve action readiness and behavior provision, and further affect individual cognition, judgment, and behavior (Lee and Choi, 2022). For example, displaying products of a tactile attribute in visual language (Park and Yoo, 2020) or verbal descriptions of target objects or extrinsic sensory cues induce the individuals to initiate their mental simulations (Lv et al., 2020), which will facilitate subsequent behavior. Therefore, mental simulation is a marketing strategy often used by marketers to attract consumers’ attention and encourage participation, thus changing consumer behavior (Choi et al., 2020).

## Hypotheses development

### The influence of metaphorical visual language haptic cues on consumers’ purchase intention

Visual language has been defined differently in many fields (Erwig et al., 2017). This study follows the definition of visual language by Kahn (1996) and Strothotte and Strothotte (2012). In a broad sense, any type of non-textual visual communication medium (including art, images, maps, and charts) can integrate visual elements into a unified communication unit, and a visual language is formed. The cognitive availability advantage of visual language comes from its picture characteristics (Blackwell, 1996). Individuals process visual language with approximately 20 billion brain neurons to help them analyze quickly and integrate relationships into visual elements in terms of visual structure (Malamed, 2009; Emanuel and Challons-Lipton, 2013), thus, people have a sufficient cognitive basis and cognitive advantages *via* visual language (Green and Petre, 1996; Blackwell et al., 2001). Visual language has long been widely used in marketing. For example, retailers design different visual languages on packaging to make people perceive their product difference (Vila and Ampuero, 2006; Vollenbroek, 2021) and to influence people’s judgment and attitude toward products (Chrysochou and Grunert, 2014; Delivett et al., 2020).

Visual structures employed in visual language include metaphor (Blackwell, 2001), hyperboles (Schilperoord and Maes, 2010), and ellipsis (Van Mulken et al., 2014). As an important auxiliary means for people to understand visual language, a metaphor compares two different visual elements, indicating when one object is similar to another one (Jeong, 2008), the “similarity” between two visual elements will be highlighted in the same visual language (Phillips and McQuarrie, 2004; Lagerwerf et al., 2012). Consumers usually carry out the best perceptual organization of different objects with parallel perception according to the similarity law and law of contiguity. Thus, the metaphor or the link of relative concepts between them can be recognized and inferred (Schilperoord et al., 2009). Existing research confirms that presenting MVL can help people construct relevant visual language processing programs in their psyche and mind, understand the meaning conveyed by visual language (Blackwell, 2001), provide people with near-authentic tactile information (Ghosh and Sarkar, 2016), and further influence people’s purchase intentions and brand attitudes toward the target product among others (Fenko et al., 2018; Margariti et al., 2019; Myers and Jung, 2019). Therefore, the two visual elements of the MVL defined in this study refer to the product element and the metaphorical element, with the product element being the target product image and the metaphorical element invoking the haptic cues in the text, both of which are metaphorically juxtaposed in the visual language display.

According to the theory of touch visualization, real haptic perception can be generated when an individual watches the intentional or unintentional touch between living or inanimate objects, thus, emerging vicarious touch experience (Keysers et al., 2004; Schirmer and McGlone, 2019). In e-commerce practice, online retailers often rely on MVL by designing relevant haptic cues in the display of the target product, such as a hand touching the product or an object with significant tactile properties, in order to express and convey the tactile properties of the target product visually and concretely. On the basis of the concept of Luangrath et al. (2022), vicarious touch is conceptualized as the touch between the haptic cues in MVL in picture display and the product, so that consumers can have a nearly real tactile experience. In this paper, haptic cues are specifically divided into hand haptic cues and object haptic cues (Ebisch et al., 2008).

Different haptic cues have different effects on consumers’ understanding of the nature and strength of online products’ tactile attributes, which leads to differences in behavioral intentions. The “placebo” effect indicates that irrelevant or non-diagnostic information will affect consumers’ judgment (Shiv et al., 2005; Brasel and Gips, 2014; Abrate et al., 2021). Therefore, attention to haptic cues in MVL can be transferred to target products to a certain extent. Consumers are able to shift this attention to some extent to the target product, evoking a mental simulation of the target product (Herrero et al., 2022), enhancing the perceived tactile attributes of the product (Biswas and Szocs, 2019), and influencing subsequent perceptions of product quality, brand attitudes, and purchase intentions (Yoganathan et al., 2019; Donato and Raimondo, 2021). Thus, when haptic cues in MVL are objects, through demonstrating haptic properties significantly related to objects, consumers can actually feel product haptic properties based on vicarious touch experience and transcendental knowledge get from haptic properties and quality and promote a positive attitude. For example, Lv et al. (2020) and Maille et al. (2020) proved that product images with haptic cues could stimulate the mental simulation and purchase intention of the subjects through the manipulation of haptic cues such as feathers, brushes, or wine glasses than straightforward product displays. From the perspective of consumers’ online environment feelings, haptic cues of heavy and soft objects affect both the warmth and ability dimensions of online retailers, thus influencing purchase intention (Jha et al., 2019). When the haptic cue is hand, i.e., when showing the image of the target product touched by the hand, with the help of metaphorical rhetorical techniques to simulate the process of exploring the tactile properties of the product by consumers themselves using their hands, this vicarious touch experience makes it a real touch experience. Lederman and Klatzky (1987) showed that the initiative and diagnostic hand exploration process (EPs) can be mapped to the tactile system and extract relevant haptic information. Additionally, in the context of observing others’ hand touch online, touching products with others’ hands leads to increased somatosensory activity in brain regions (Basso et al., 2018) and vicarious experiencing (Luangrath et al., 2022). Studies in cognitive psychology and consumer behavior have shown that observing hand haptic cues and product touch could stimulate vicarious touch experience. The process of automatically simulating others’ hand-touch actions appears psychologically and behaviorally (Liu et al., 2018), even if the subjects rationally judge that the behavior does not come from themselves (Wegner et al., 2004). This vicarious sensory experience also helps to infer its sensory characteristics and obtain sensory information about products (Pino et al., 2020), thereby enhancing purchase and payment intention (Luangrath et al., 2022).

Gkiouzepas and Hogg (2011) proposed conceptual tension to explain the contiguity of metaphorical conceptual links, i.e., the degree of correlation between two metaphorical objects (concepts). Our study refers to the conceptual tension between the two metaphorical objects of the haptic cue and the target product. When the metaphorical targets displayed in visual language are closely related to each other, such as when haptic cues are closely linked to the target product in terms of tactile properties, the metaphorical degree of conceptual tension may affect their metaphorical quality (McCabe, 1983), i.e., the smaller the conceptual tension between two metaphorical targets (concepts), the more familiar the individual is to the source domain. Therefore, compared to two metaphorical objects with high conceptual tension two metaphorical targets, with less conceptual tension can help individuals perceive metaphorical representation from the whole and enhance their episodic memory of metaphorical pictures (Gurguryan et al., 2021), and the quality of perceived visual metaphors will be better (Pollock, 2020). Thus, compared with haptic cues of objects, haptic cues of hands and products have closer conceptual links and less conceptual tension. This is because the hand is one of the most sensitive parts of the human body, as suggested by Penfield touch dwarf theory (Sekuler and Blake, 1994), and it is the haptic “window” for exploring objects (Klatzky et al., 1993). In addition, touching products are very active in nature. Therefore, people are familiar with how to judge and experience the tactile properties of products through hand touch (Luangrath et al., 2022). Studies have shown that when people see the virtual hand or real rubber hand touching objects, they begin to feel that the virtual hand (Moseley et al., 2008; Slater et al., 2009) and the rubber hands (Ehrsson, 2020) are their own hands, and “tactile transmission” phenomenon occurs, which reduces consumers’ distance with the rubber hand or virtual hand and blurred the boundary with the body, thus, promoting vicarious tactile experience. Therefore, people are more familiar with the haptic cues of the hands than with haptic cues about objects. Then the conceptual tension of metaphor containing hand haptic cues is smaller than object haptic cues, and people are more likely to accept and understand these tactile attributes and experiences conveyed by these hand haptic cues in MVL. To sum up, the following hypothesis is proposed:

H1: Online products containing haptic cues in MVL, compared with haptic cues of objects, haptic cues of hands can lead to more positive purchase intention.

### The mediating effect of mental simulation

It is easier to activate rich sensory information stored in working memory through metaphor presentation, such as recently perceived stimulus images, or sensory images possibly extracted from long-term memory (Abaidi et al., 2020). Thus, MVL can convey the richness and vividness of tactile information, and visual language containing rich sensory information is more effective than other stimuli in evoking mental simulation (Lee and Gretzel, 2012; Liu et al., 2019; Lee and Choi, 2021). Embodied simulation neuroscience model indicates that individuals conceptualize observed things based on their own body–related perceptual experiences (Gallese, 2005; Tan, 2020) and create cognitive psychological shortcuts. Therefore, both hand haptic cues and object haptic cues contain abundant tactile information and can represent consumers’ apriority tactile experience. Forming from the metaphor of structure mapping instead of tactile experience, the vicarious tactile experience can help consumers quickly get conceptual cognition of online product tactile properties, thus, effectively stimulating mental simulations (Chen and Lin, 2021; Yim et al., 2021).

Mental simulation can be distinguished into two elaboration types: process simulation and outcome simulation (Zhao et al., 2007). Process simulation involves imagining the process, emphasizing the actions necessary to achieve the results, and encouraging the formation of plans through step-by-step stories or narratives. People who are more focused on process simulation are better at reinforcing product knowledge and will concentrate more on relatively precise means (Rennie et al., 2014) and techniques to accomplish their objectives (Agrawal and Wan, 2009). While outcome simulation involves people’s imagination of the outcomes of events, emphasizing the outcomes that people expect to occur when they imagine achieving their goals (Taylor et al., 1998; Wu et al., 2021). People who are more focused on outcome simulation are more likely to exhibit impulsive behavior in reaction to stimuli (Rennie et al., 2014), and they concentrate on outcomes that are vague and abstract (Agrawal and Wan, 2009). Process simulation and outcome simulation facilitate the visualization of product adoption decisions (Castaño et al., 2008), focusing on the “how” and “why” modes of thinking, respectively. When the type of mental simulation matches the processing mode, the target, or the memory of previously stored information, mental simulation can play the most concrete role (Zhao et al., 2011). Haptic cues in stocktickerMVL facilitate each of these two mental simulations. Process simulation focuses on the process of enjoying the experience. When consumers attach importance to the experience process of products, they tend to touch and explore with their hands. Hand cues in stocktickerMVL can maximize the stimulation of process simulation (Yim et al., 2021), such as the softness of towels. While outcome simulation focuses on the benefits of the tactile experience. When consumers think highly of product outcome information, object cues in MVL can maximize outcome simulation (Xie et al., 2016).

Mental simulation of previous experience triggered by external cues can influence consumers’ expectations of the product, experience, and evaluation (Papies et al., 2017). Persuasive information that evokes stronger imagery, such as information containing multisensory modality, can strongly induce mental simulation, thereby initiating strong product attitudes and behavioral intentions (Chen and Lin, 2021). Thus, haptic cues in MVL of products can effectively stimulate process simulation and outcome simulation (Xie et al., 2016) and influence preferences, attitudes, and behavioral intentions (Chang, 2012; Petit et al., 2017, 2018). Therefore, the following hypothesis is proposed:

H2: Mental simulation plays a mediating role in the influence of haptic cues in the MVL of products on consumers’ purchase intention. Among them, process simulation mediates hand haptic cues and purchase intention, and outcome simulation mediates object haptic cues and purchase intention.

### The moderating effect of product type

Based on the division of product types in existing studies, products are divided into functional products and sensory-social products (Bettiga et al., 2020). Functional products are instrumental and practical, and consumers need to think, analyze, and process the attributes of the products, including products with functional purposes (such as electronic products and water cups) (Zhu and Meyer, 2017), and their potential purchase motivation is the material functional attributes maximization (Daugherty et al., 2008). This kind of product is the most suitable for collecting geometric information such as shape attributes and haptic information such as weight (McCabe and Nowlis, 2003; Jang and Ha, 2021). With sensory-social products, consumers focus on self-expression and sensory pleasure and usually attach personal emotional factors. When the most important sensory attribute of sensory-social products is touch, it is difficult for consumers to evaluate their haptic attributes before purchasing. Such as texture dimension-related information, which needs to be verified by real touch exploration (Casasanto, 2009; Milhau et al., 2017; Ranaweera et al., 2021). Therefore, according to the different roles of sensory information such as MVL haptic cues in functional products and sensory-social products, products can be divided into tactile functional products and tactile experiential products (Bettiga et al., 2020; Karangi and Lowe, 2021). Processing fluency is defined as the ease with which information is processed (Alter and Oppenheimer, 2009). The theory of processing fluency indicates that individuals automatically form self-perception of the difficulty and fluency of processing information (Shapiro, 1999; Murphy et al., 2022), allowing individuals to form self-preference judgments and emotional responses to processed information (Carr et al., 2016), which are primarily related to people’s mental conceptual fluency and perceptual fluency (Lee and Labroo, 2004; Dreisbach and Fischer, 2011). When people develop higher mental fluency and perceptual fluency for processed information, it is beneficial for people to select, evaluate, and make corresponding purchases of processed information (Winkielman and Berridge, 2003; Alter and Oppenheimer, 2006; Novemsky et al., 2007). When the MVL information is consistent with the relevant attributes of the product, the processing fluency of consumers will be improved and they can quickly process the corresponding sensory information (Chen and Lin, 2021), which can influence subsequent behavior. Therefore, tactile functional products highlight instrumental tactile information through tactile interaction between objects’ haptic cues in MVL and tactile functional products, so that consumers can quickly identify tactile attributes, extract tactile information, produce outcome simulation, and promote their purchase intention without realizing it. For tactile experiential products, tactile information is collected through hand-touch exploration (Bergmann Tiest et al., 2012) and familiarity is enhanced (Yim and Yoo, 2020). Tactile vicarious experience is provided to reduce perceptual risks and enhance a pleasant shopping experience (Cano et al., 2017). Thus, the interaction between hand haptic cues and tactile experiential products can meet the demand for touching products, which is in favor of generating process simulation and promoting purchase intention. In conclusion, the following hypothesis is proposed:

H3: Product type plays a moderating role in the influence of haptic cues in MVL on consumers’ purchase intention. Among them, tactile experiential (tactile functional) products in significant tactile attribute products display using hand (object) haptic cues will lead to positive purchase intention.

**FIGURE 1:**
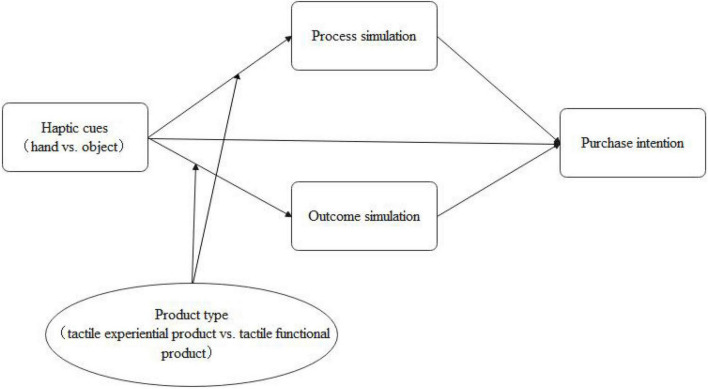
The theoretical model.

The theoretical model of this paper is shown in Figure 1.

## Methodology

### Study 1: Online shopping platform real data analysis

#### Pre-study

Before starting the experiment, all the products needed for the experiment should be determined. In the first step, subjects were asked to evaluate product attributes considered when purchasing the product and score the importance of tactile and visual information in product evaluation and decision-making. Combining the product categories used in the previous studies, 30 products sold online were selected first. Referring to the experimental procedure of Balaji et al. (2011) on the pre-test of product types, 40 undergraduates from a university in China were invited. It was assumed that the importance of tactile information (TI) and visual information (VI) was rated from extremely unimportant (1) to extremely important (5) according to a Likert 5-point scale in the decision of purchasing these products. Statistical results showed that 12 kinds of products were tactile diagnostic products, including pyjamas (*N* = 40, TI = 4.90, VI = 3.85, *p* < 0.001), dumbbells (*N* = 40, TI = 4.48, VI = 2.80, *p* < 0.001), blankets (*N* = 40, TI = 4.90, VI = 4.10, *p* < 0.001), gloves (*N* = 40, TI = 4.85, VI = 3.80, *p* < 0.001), pinch meter (*N* = 40, TI = 4.48, VI = 2.50, *p* < 0.001), and scarf (*N* = 40, TI = 4.78, VI = 4.50, *p* = 0.020).

In the second step, tactile diagnostic products were further divided into tactile functional products and tactile experiential products. Fifty undergraduates from a university in China were invited to measure whether they pay attention to tactile functional information (TFI) or tactile experiential information (TEI) when purchasing 12 tactile diagnostic products using a Likert 5-point scale. Statistical results showed that blanket (*N* = 50, TFI = 4.04, TEI = 4.50, *p* = 0.021), pyjamas (*N* = 50, TFI = 3.82, TEI = 4.38, *p* = 0.010), and scarf (*N* = 50, TFI = 3.90, TEI = 4.42, *p* = 0.013) were tactile experiential products; dumbbells (*N* = 50, TFI = 4.28, TEI = 3.66, *p* = 0.007) and pinch meter (*N* = 50, TFI = 4.42, TEI = 3.90, *p* = 0.010) were tactile functional products; and gloves (*N* = 50, TFI = 4.26, TEI = 4.06, *p* = 0.274) were hybrid products.

Therefore, gloves, scarves, and pinch meter were selected as the products for secondary data collection in Study 1. Blankets were selected as the experimental products in Study 2, and pyjamas and dumbbells were selected as the experimental products in Study 3.

#### Formal experiment

To test the relationship between consumers’ haptic cues when shopping and their purchasing behavior in online retail, this study selected gloves (tactile functional and tactile experiential mixed product), scarf (tactile experiential product), and pinch meter (tactile experiential product). Nearly 13,000 online purchase data were intercepted from the Taobao platform to inquiry whether the lack of haptic sense can be compensated by the touch between haptic cues and the product, under the premise of controlling other conditions.

##### Data collection

“Gloves/scarves/pinch meter” were entered into the search bar of the Taobao platform, and 4,630 gloves, 4,567 scarves, and 4,534 pinch meter were available. In order to control the influence of other variables on sales as much as possible and verify the influence of haptic cues on sales, this study chose the cover display pictures on the shopping page. The purchase page presented information such as price, product description, picture, and store name. Therefore, except for the influence of haptic cues, the price and the number of online reviews were also included in the study.

##### Coding

The criteria for pictures with or without haptic cues were determined. For example, for gloves, the use of models’ hands to display gloves or placing cotton and other objects beside them was considered as having haptic cues; otherwise, there were no haptic cues (0 = no haptic cues, 1 = have haptic cues). Scarves and pinch meter were coded in the same way.

##### Results

Taking the logarithm of the price in the product picture and the number of online reviews into the model to eliminate the influence of different dimensions of each variable (Huang et al., 2019; Lv et al., 2020):


Ln(Quantity)=C+β⋅1Ln(Price)+β⋅2Ln(Comments)+β⋅3Cues+ε

ε is the random error term, C is constant, Quantity is product sales volume, Price is product price, Comments is the online comment, and Cues is haptic cues.

Linear regression was performed on the above variables; the results of these three products will be shown in the following tables (Tables 1–3). Taking glove (hybrid product) as an example, three variables had a significant impact on sales [*R*^2^ = 0.757, *F*(3,4627) = 4816.900, *p* < 0.001]. Specifically, the glove pictures with haptic cues had a positive correlation and significant impact on its sales [*B* = 0.407, *t*(4628) = 8.143, *p* < 0.001], i.e., compared with no haptic cues, the glove pictures with haptic cues had higher sales. In addition, the glove price had a negative correlation and significant impact on the sales volume [*B* = –0.191, *t*(4628) = –9.367, *p* < 0.001]. The higher the glove price, the lower the sales volume. The number of online reviews of gloves had a positive correlation with the sales volume [*B* = 0.960, *t*(4628) = 114.871, *p* < 0.001]. The more online reviews accumulated, the higher the sales volume. The same analysis was used for both the scarf and pinch meter.

**TABLE 1:** Results of haptic cues influence in glove presentation.

Variables	Unstandardized coefficients	Standardized coefficients	*t*	Sig.
C	1.509		16.384	0.000
Ln(Price)	–0.191	–0.070	–9.367	0.000
Ln(Comments)	0.960	0.855	114.871	0.000
Cues	0.407	0.059	8.143	0.000
*R* ^2^	0.757			
*R*^2^ (adjusted)	0.757			
Sig. F	0.000			
*F*	4816.900			

**TABLE 2:** Results of haptic cues influence in scarf presentation.

Variables	Unstandardized coefficients	Standardized coefficients	*t*	Sig.
C	1.771		21.858	0.000
Ln(Price)	–0.105	–0.060	–6.955	0.000
Ln(Comments)	0.856	0.811	94.792	0.000
Cues	0.101	0.022	2.595	0.009
*R* ^2^	0.683			
*R*^2^ (adjusted)	0.683			
Sig. F	0.000			
*F*	3283.244			

**TABLE 3:** Results of haptic cues influence in pinch meter presentation.

Variables	Unstandardized coefficients	Standardized coefficients	*t*	Sig.
C	0.641		8.354	0.000
Ln(Price)	–0.051	–0.019	–2.282	0.023
Ln(Comments)	1.030	0.796	92.766	0.000
Cues	0.522	0.105	12.276	0.000
*R* ^2^	0.689			
*R*^2^ (adjusted)	0.689			
Sig. F	0.000			
*F*	3345.790			

##### Discussion

A secondary data study showed that haptic cues in product pictures had a significant impact on consumer purchasing behavior. Products were sold more when product pictures included haptic cues. Otherwise, it led to lower sales. This was partly because the haptic cues in the pictures helped consumers to simulate the products to some extent when they bought products. However, Study 1 did not distinguish and classify haptic cues, so in the following experiments, this paper further distinguishes different types of haptic cues and verifies the influence of haptic cues on purchase intention.

### Study 2: Main and mediating effect test

#### Pre-study

**FIGURE 2:**
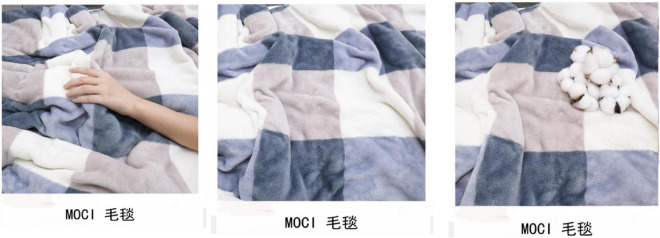
Study 2 stimulus materials.

First, the design of the images in the experiment should be as consistent as possible, such as image size, color, subject, description, and other visual elements consistent with minimizing confusion about other observable factors. The only difference between these pictures differed in the type of haptic cues. Among them, the resolution of the product pictures was 825*1492, and the opacity was 100%. The only difference between pictures of each group was the haptic cue included. Three groups of experimental stimulus materials were designed to display different haptic cues, namely, hand haptic cue, object haptic cue, and non-haptic cue. Among them, the hand haptic cue was that a model’s hand was touching the product. Object haptic cue was that an object with highly significant tactile attributes was touching a product to highlight tactile attributes. The display without haptic cue was a separate product display and it was designed as the control group. As shown in Figure 2.

Referring to the pre-test of process simulation and outcome simulation of Xie et al. (2016), subjects were determined that they were capable of process and outcome simulation of the product. Referring to the mental simulation scale developed by Xie et al. (2016), a Likert 7-point scale of “not at all” (1) and “to a great extent” (7) was used. Scale details in Appendix Table 1. In the experiment, subjects were asked to watch product pictures (hand haptic cue vs. object haptic cue vs. control group: no haptic cue) and reported their ability to simulate the process of using the product and the degree of simulated enjoyment after using the product (such as simulating the touch of the product, feeling its weight, and texture softness). At the same time, with reference to metaphor control, a Likert 7-point scale of “not at all important” (1) and “extremely important” (7) was used to ask subjects to report whether the product description they viewed was “straightforward and factual” or “figurative and abstract.”

A total of 117 undergraduates from a university in China were randomly assigned to the above three experimental groups. An ANOVA revealed a significant effect on mental simulation [*F*(2,114) = 14.175, *p* < 0.001, η^2^ = 0.199], with hand haptic cue (*M*_hand_ = 5.73, *SD* = 0.77) significantly increasing mental simulation relative to the object haptic cue (*M*_object_ = 5.14, *SD* = 0.84, *p* = 0.007) and no cue (*M*_control_ = 4.58, *SD* = 1.18, *p* < 0.001). These results indicated that the subjects could perform a greater degree of mental simulation on the hand haptic cue. There was less conceptual tension in the hand haptic cue, and subjects were suitable and capable to complete the related cognitive tasks that required the initiation of mental simulation.

An ANOVA revealed a significant effect on metaphor control [*F*(2,114) = 47.343, *p* < 0.001, η^2^ = 0.454], with hand haptic cue (*M*_hand_ = 4.74, *SD* = 0.93) significantly increasing metaphor control relative to the object haptic cue (*M*_object_ = 4.20, *SD* = 1.11, *p* = 0.015) and no cue (*M*_control_ = 2.74, *SD* = 0.75, *p* < 0.001). These results indicated that subjects perceived a higher degree of metaphor in the hand haptic cue group. There was less conceptual tension in the hand haptic cue group, and the manipulation of metaphorical picture design in the experimental stimulus material was successful in this study.

#### Formal experiment

##### Experimental design and process

The purpose of Study 2 was to explore the potential and mechanism of the haptic cue in achieving tactile compensation in online product presentations. The formal experiment was designed as a single-factor experiment between subjects. The display of haptic cues included: hand haptic cue vs. object haptic cue vs. control group: no haptic cue and the experimental product was a blanket (tactile experiential product). Using G*power software, it was calculated that at a significance level of 0.05 and a moderate effect size (*f* = 0.25), the total sample size predicted to reach a statistical power level of 80% was at least 159. Thus, the planned sample size was 190. Due to epidemic and subject time conflicts, finally, 165 undergraduates from a university in China were invited and randomly divided into three groups (56, 54, and 56, respectively, including 90 women). All subjects were aged 18–25 and had online shopping experiences.

According to the experimental design of Van Mulken et al. (2014), three experimental situations were designed, and a virtual product named “MOCI” was introduced. The product display page was forced to be exposed for 20 s to simulate the online shopping environment. Only after the retention time were subjects allowed to open the next page and fill in the subsequent test objects. Subjects were introduced to a simulated online shopping situation, where they were all asked to imagine themselves shopping online. After browsing the product display page, they were required to fill in the process simulation scale (α = 0.853), outcome simulation scale (α = 0.881) [adapted from Elder and Krishna (2012) and Xie et al. (2016)], purchase intention scale (α = 0.863), and other research scales as well as fill in relevant demographic variables. Scale details in Appendix Table 1.

##### Results

###### Manipulation test

ANOVA results showed there were significant differences in the degree of metaphorical expression among the three groups ([*F*(2, 163) = 84.237, *p* < 0.001, η^2^ = 0.508]. There were significant differences in the degree of metaphorical control between the hand haptic cue (*M*_hand_ = 4.71, *SD* = 0.75) and object haptic cue (*M*_object_ = 4.38, *SD* = 0.78, *p* = 0.023), and with the control group (*M*_control_ = 2.96, *SD* = 0.74, *p* < 0.001). This indicated successful manipulation of haptic cues.

###### Main effect test

ANOVA results showed that haptic cues display had a significant effect on consumers’ purchase intention [*F*(2,163) = 21.476, *p* < 0.001, η^2^ = 0.209]. Participants had a higher level of purchase intention for the product with hand haptic cue (*M*_hand_ = 4.98, *SD* = 0.80) relative to the object haptic cue (*M*_object_ = 4.24, *SD* = 0.85, *p* < 0.001) and no cue (*M*_control_ = 3.78, *SD* = 1.23, *p* < 0.001). Further *post hoc* tests yielded significant differences between all three groups (all *p* < 0.05). These effects are not qualified by age (*p* = 0.470) or gender (*p* = 0.673). Thus, H1 was verified.

###### Mediating effect test

**FIGURE 3:**
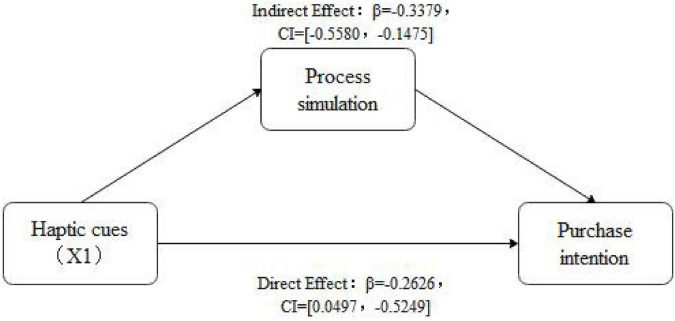
The mediating effect of process simulation.

According to the mediating effect analysis procedure proposed by Zhao et al. (2010), Bootstrap methods proposed by Preacher et al. (2007) and Hayes (2013) were applied to examine the mediating effects. Mediation analysis model 4 was selected, and the sample size was 5,000. The sampling method was the non-parametric percentile method with selection bias correction under 95% confidence intervals. Purchase intention was the dependent variable, haptic cues were the independent variable, and process simulation and outcome simulation were the dual mediating variables. The independent variable of haptic cues was multiple categorical variables, thus, it was re-coded. Two dummy variables X1 and X2 were added with the control group as the benchmark X1 (control vs. hand) and X2 (control vs. object) were defined as independent variables. Results showed that process simulation had a mediating effect (β = –0.3379, *SE* = 0.1044, CI = [–0.5580, –0.1475]), with X1 (hand vs. control) as the independent variable, process simulation the mediating variable, and purchase intention the dependent variable. The specific results are shown in Figure 3.

###### Exclusion of other explanations

In conducting the above mediation test, it was also tested whether the process simulation and the outcome simulation could play a mediating role when the independent variable was X2 and whether the outcome simulation could play a mediating role when the independent variable was X1. When X2 (object vs. control) was the independent variable, process simulation was the mediating variable, and purchase intention was the dependent variable, process simulation had no significant mediating effect (β = –0.1583, *SE* = 0.0959, CI = [–0.3570, 0.0122]). When X2 (object vs. control) was the independent variable, outcome simulation was the mediating variable, and purchase intention was the dependent variable, outcome simulation had no significant mediating effect (β = –0.2004, *SE* = 0.1551, CI = [–0.5229, 0.0854]). Taking X1 (hand vs. control) as the independent variable, outcome simulation as the mediating variable, and purchase intention as the dependent variable, it was found that there was no significant mediating effect of outcome simulation (β = –0.1113, *SE* = 0.0642, CI = [–0.2472, 0.0081]).

##### Discussion

The results of the study’s second main effect support H1 that MVL representations of haptic cues positively influence consumers’ purchase intentions. Specifically, the presentation of haptic hand cues elicited more positive purchase intentions compared to object haptic cues and no haptic cues. The new contribution of our study compared to (Lv et al., 2020; Maille et al., 2020) is that it focuses on the design of haptic cues and extends on the metaphorical structure of visual language from mental simulation theory, confirming that not only the presence of object haptic cues can play a tactile compensatory role but also hand haptic cues can be used as a haptic cue to achieve tactile compensation and enhanced purchase intention.

Mediating effect analysis of Study 2 showed that, even when the consumer was buying online and could not have direct experience of the sense of touch, haptic cues could enhance the degree of consumer mental simulation. Specifically, process simulation mediated hand haptic cue and purchase intention, and the results of outcome simulation’ mediating effect had not been confirmed, leading to H2 being partly verified. This study believed that the reason for H2 partly verified may be that consumers tend to feel and enjoy the soft and warm blanket by hand but outcome simulation cannot mediate this process. Therefore, there might be a boundary mechanism leading to this result. If there is a boundary mechanism, can the conclusion in H1 that hand haptic cues can induce more positive purchase intention than object haptic cues be proved again? Therefore, on the basis of Study 2, Study 3 further expands the experimental validity, expands the offline experiment to each online group of subjects, and explores the influence of its boundary mechanism.

### Study 3: Moderating effect test

#### Pre-study

According to the pre-experiment of product type, the coral-wool pyjama was determined as a tactile experiential product, and the dumbbell was determined as a tactile functional product.

**FIGURE 4:**
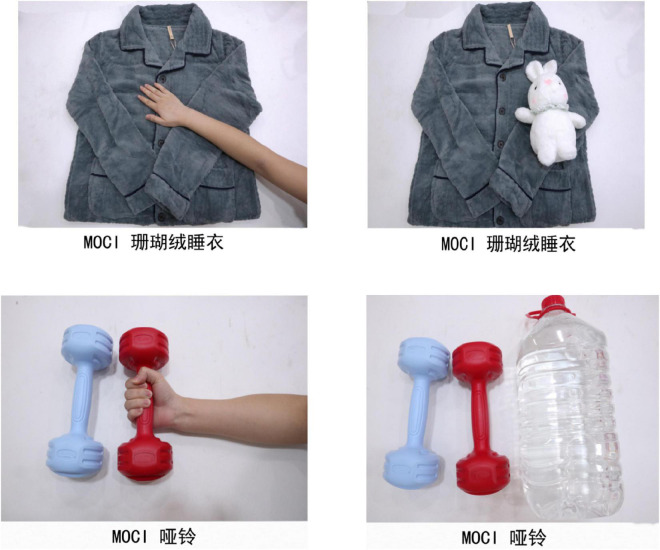
Study 3 stimulus materials.

Similar to the experimental design of Study 2, to ensure that experimental pictures of two products were consistent with other factors except for haptic cues, a single-factor inter-group design was adopted. All subjects were divided into the hand haptic cue group and an object haptic cue group. As shown in Figure 4. For the experimental products of coral velvet pyjamas, 90 questionnaires were distributed through the Credamo platform, and 83 valid questionnaires were randomly divided into two experimental groups. Results of ANOVA showed that the degree of metaphor control of the hand haptic cue (*M*_hand_ = 4.17, *SD* = 0.79) was significantly higher than that of the object haptic cue [*M*_object_ = 3.87, *SD* = 0.56; *F*(1,81) = 4.426, *p* = 0.039]. Similarly, for dumbbell, 90 questionnaires were distributed through the Credamo platform, and 84 valid questionnaires were randomly divided into two experimental groups. Results of ANOVA showed that the degree of metaphorical perception of object haptic cue (*M*_object_ = 4.21, *SD* = 0.71) was significantly higher than that of hand haptic cue [*M*_hand_ = 3.86, *SD* = 0.63; *F*(1,82) = 5.679, *p* = 0.019]. It indicated that the metaphorical control of the two groups was successfully manipulated and expressed.

Participants who completed the metaphorical control pre-experiment continued to participate in the mental simulation pre-experiment. For the experimental products of pyjamas, the results of ANOVA showed that the degree of mental simulation of the hand haptic cue (*M*_hand_ = 5.58, *SD* = 0.77) was significantly higher than that of the object haptic cue [*M*_object_ = 5.32, *SD* = 0.89; *F*(1,81) = 8.496, *p* = 0.005]; For dumbbell, the degree of mental simulation of the object haptic cue (*M*_object_ = 5.42, *SD* = 0.66) was significantly higher than that of the hand haptic cue [*M*_hand_ = 4.88, *SD* = 1.32; *F*(1,82) = 5.530, *p* = 0.021]. These results indicated that the participants had less conceptual tension between pyjamas (dumbbell) and hand haptic cue (object haptic cue) and were able to complete related cognitive tasks requiring mental simulation.

#### Formal experiment

##### Experimental design and process

Study 3 was a two-factor group design: 2 (haptic cue: hand vs. object) × 2 (product type: tactile functional product: dumbbell vs. tactile experiential product: pyjamas). The experimental process of Study 3 was similar to Study 2. A virtual brand named “MOCI” was introduced. After glancing over the product display page, participants filled in the process simulation (α = 0.897), outcome simulation (α = 0.902), purchase intention (α = 0.927) scales, and demographic variables. Using G*power software, it was calculated that at a significance level of 0.05 and a moderate effect size (*f* = 0.25), the total sample size predicted to reach a statistical power level of 90% was at least 171. So 220 questionnaire was distributed through the Credamo platform, and 198 valid questionnaires were recovered (101 women and 97 men, with an average age of 28 years), 92% of the subjects had more than 3 years of online shopping experience. All the subjects were randomly assigned to four experimental groups (50, 50, 50, and 48, respectively).

##### Results

###### Test of metaphorical control manipulation

In the coral wool pyjamas group, ANOVA results showed that the metaphorical perception degree of the hand haptic cue (*M*_hand_ = 4.09, *SD* = 0.72) was significantly higher than that of object haptic cue [*M*_object_ = 3.74, *SD* = 0.67; *F*(1,98) = 6.212, *p* = 0.014]. In the dumbbell group, ANOVA results showed that the metaphorical perception degree of object haptic cue (*M*_object_ = 3.66, *SD* = 0.69) was significantly higher than that of hand haptic cue [*M*_hand_ = 3.41, *SD* = 0.50; *F*(1,96) = 4.369, *p* = 0.039], indicating successful metaphorical manipulation of the two groups.

###### Main effect test

**FIGURE 5:**
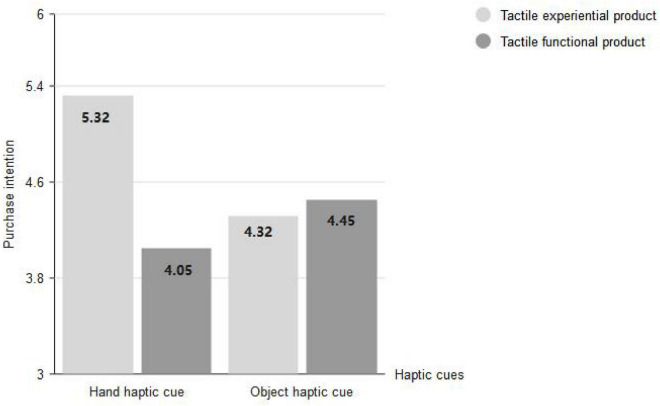
Interaction effects between haptic cues and product types.

The purchase intention was used as the dependent variable to test whether there were interaction effects between haptic cues and product types. ANOVA results showed that the interaction effect between product types and haptic cues was significant (*F* = 26.384, *p* < 0.001, η^2^ = 0.120), the main effect of product types was significant (*F* = 17.077, *p* < 0.001, η^2^ = 0.081), and the main effect of haptic cues was also significant (*F* = 4.707, *p* = 0.031, η^2^ = 0.024). A simple effect test further found that when products were tactile experiential products, the main effect of the haptic cue was significant, and the hand haptic cue (*M*_hand_ = 5.32, *SD* = 1.03) got higher purchase intention than the object haptic cue [*M*_object_ = 4.32, *SD* = 0.83; *F*(1,98) = 28.949, *p* < 0.001]. When products were tactile functional products, the main effect of the haptic cue was significant, and the haptic cue of the object (*M*_object_ = 4.45, *SD* = 1.06) caused higher purchase intention than the haptic cue of hand [*M*_hand_ = 4.05, *SD* = 0.94; *F*(1,96) = 4.072, *p* = 0.046], which is shown in Figure 5. These effects are not qualified by age (*p* = 0.087) or gender (*p* = 0.190).

###### Mediating effect test

Process simulation was used as the dependent variable to test whether there were interaction effects between haptic cues and product types. ANOVA results showed that the interaction effect between product types and haptic cues was significant (*F* = 3.925, *p* = 0.049, η^2^ = 0.020), and the main effect of product type was significant (*F* = 23.191, *p* < 0.001, η^2^ = 0.107), that is to say, tactile experiential products (*M*_experiential_ = 5.17, *SD* = 0.95) caused more process simulation than tactile functional products [*M*_functional_ = 4.50, *SD* = 1.06; *F*(1,196) = 21.964, *p* < 0.001]. The main effect of haptic cues was also significant [*F* = 8.175, *p* = 0.005, η^2^ = 0.040]. Thus, the hand haptic cue (*M*_hand_ = 5.03, *SD* = 1.10) caused more process simulation than the object haptic cue [*M*_object_ = 4.64, *SD* = 1.00; *F*(1,196) = 7.096, *p* = 0.008]. In order to test the mediating effect of process simulation, this study drew upon the mediation analysis procedure proposed by Zhao et al. (2010), and the adjusted mediation analysis model 8 proposed by Preacher et al. (2007) and Hayes (2013) to conduct the Bootstrap mediation variable test. The sample size was set to 5,000, and under the 95% confidence interval, process simulation did mediate the interaction between haptic cues and product types on purchase intention. The mean value of the interaction effect size was 0.1573, and the confidence interval of the Bootstrap test was [0.0035, 0.3215], excluding 0, indicating the existence of moderated mediation effect. To be specific, when the product was the tactile experiential product, the mean value of the indirect effect size was 0.1922, and the confidence interval of the Bootstrap test was [0.0675, 0.3382], excluding 0, indicating that the indirect effect was significant. These results showed that when browsing tactile experiential products, the mediating effect of process simulation existed, as shown in Figure 4.

**FIGURE 6:**
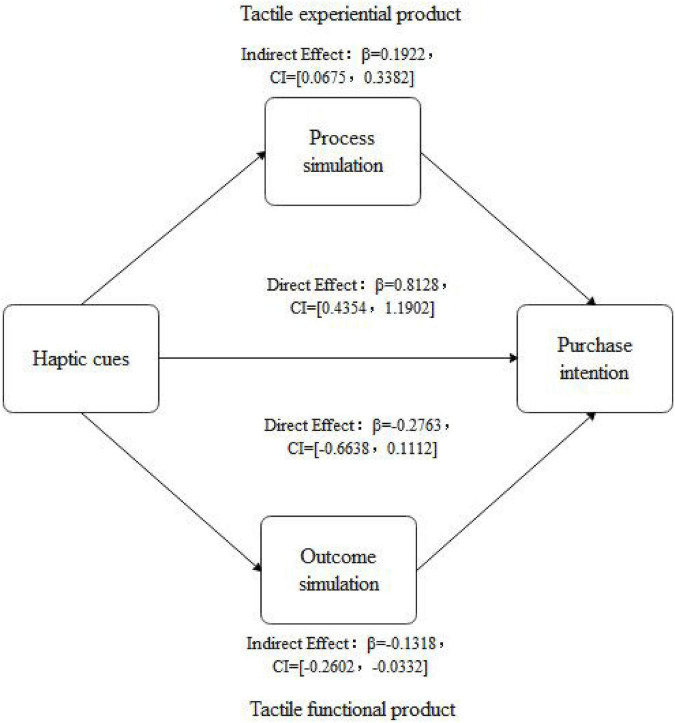
Mediating effect of process simulation (tactile experiential product) and outcome simulation (tactile functional product).

Similarly, the outcome simulation was tested again as the dependent variable. ANOVA results showed that the interaction effect between product types and haptic cues was significant (*F* = 4.435, *p* = 0.036, η^2^ = 0.022), and the main effect of product types was significant (*F* = 5.291, *p* = 0.022, η^2^ = 0.027). Tactile functional products (*M*_functional_ = 5.08, *SD* = 1.04) caused higher outcome simulation than tactile experiential products [*M*_experiential_ = 4.73, *SD* = 1.22; *F*(1,196) = 4.880, *p* = 0.028]. The main effect of haptic cues was significant (*F* = 5.799, *p* = 0.017, η^2^ = 0.029). Object haptic cues (*M*_object_ = 5.09, *SD* = 1.06) generated higher outcome simulation than hand haptic cues [*M*_hand_ = 4.72, *SD* = 1.19; *F*(1,196) = 5.377, *p* = 0.021]. Similarly, to test the mediating effect of outcome simulation, outcome simulation did mediate the interaction between haptic cues and product types on purchase intention. The mean value of the interaction effect size was 0.1230, and the confidence interval of the Bootstrap test was [0.0053, 0.2961], excluding 0, indicating the existence of the moderated mediating effect. Specifically, when the product was the tactile functional product, the mean value of the indirect effect size was –0.1318, and the confidence intervals of the Bootstrap test were [–0.2602, –0.0332], respectively. This interval did not include 0, indicating that the indirect effect was significant. These results suggested that when browsing tactile functional products, the mediating effect of outcome simulation existed, as shown in Figure 6.

###### Ruling out other explanations

In this study, the moderated mediation test was conducted, and other conditions were also tested for the absence of mediation. For the tactile functional product, the mean indirect effect of process simulation was 0.0349, and the confidence interval of the Bootstrap test was [–0.0845, 0.1847], including 0. For the tactile experiential product, the mean value of the simulated indirect effect of outcome simulation was –0.0088, and the confidence interval of the Bootstrap test was [–0.1009, 0.8980], including 0.

##### Discussion

Study 3 aimed to explore the boundary mechanism of the influence of haptic cues in MVL on mental simulation and purchase intention. Results of Study 3 supported H3 and H2 and answered the applicable boundary conditions for H1 discussed in Study 2. The vicarious touch effect produced by haptic cues in MVL has the effect of boundary mechanisms, and not all types of products achieve optimal results using hand haptic cues, which is consistent with our main point. Also, this result fills the previous study, where Luangrath et al. (2022) chose clothing as the product type and did not explore the product type in depth. Therefore, the results of our Study 3 can be used as a complement. That is, for haptic experiential products, designing hand haptic cues can achieve the best results. For haptic functional products, on the other hand, they should be designed with object haptic cues rather than hand haptic cues.

## Findings and discussion

### Conclusion

Based on one secondary data study and two scenario simulation experiments, this paper drew the following conclusions: First, haptic cues in MVL could promote consumers’ purchase intention of tactile salient attributes products, and process simulation and outcome simulation played different mediating roles. Specifically, compared with the use of object haptic cues and non-haptic cues, the use of hand haptic cues in product display could produce a higher degree of process simulation and improve purchase intention. However, when object haptic cues were displayed, consumers would get a higher degree of outcome simulation, and then their purchase intention was improved. Second, the influence of haptic cues in MVL on consumers’ purchase intention and mental simulation had a boundary mechanism, and product types played a moderating role in it. Specifically, only the display of hand haptic cues with the tactile experiential product could enhance consumers’ purchase intention, and process simulation played a mediating role. For tactile functional products, only when displaying object haptic cues could outcome simulation play the role of mediation, so as to promote consumers’ purchase intention.

### Theoretical contributions

First, there was little literature examining the tactile compensation effect in terms of haptic cues in MVL. This paper combined the visual structure of metaphors in visual language with conceptual tension as a criterion for judging MVL to investigate consumer online tactile compensation. Based on visual language, this study proved the idea of Yim et al. (2021) and Luangrath et al. (2022) that observing another person’s hand touch also promotes tactile perception as well as product attitudes at the first step. From a metaphorical rubric conceptual tension perspective, this paper complemented Gkiouzepas and Hogg’s (2011) study that the conceptual tension of haptic cues had a positive impact on consumers’ attitudes, thus compensating for consumers’ missing tactile sensations. Thus, no matter from the point of conceptual tension or haptic cues in MVL, the suggestion of haptic cues in MVL was helpful to shorten the distance between consumers and products. It linked consumers and products from visual and tactile sensory dimensions to achieve the effect of communication between the two.

Second, consumers in the Internet context tend to obtain reliable information by observing others’ experiences and sensing sensory attributes (O’Donnell and Evers, 2019). At this time, the concept of touch visualization comes into being. While studies on touch visualization were still in the stage of physiological research (Sathian, 2016), there was a lack of empirical studies in the field of marketing. This paper differs from previous studies on the physiological stages of touch visualization by applying touch visualization theory to marketing field research. It investigated the role of touch visualization from the perspective of haptic cues in MVL, showing that adding haptic cues to MVL can significantly improve consumers’ tactile perception of products, thus compensating for consumers’ missing tactile sensation, promoting purchase intention, and enriching the research on touch visualization in the marketing field.

Finally, this paper focused on other pre-influencing factors that could stimulate mental simulation, such as haptic cues. Different from previous studies generally showing that clear and vivid product descriptions accompany this degree of sensory stimulation and activation (Yoon et al., 2021). This physiological response enhances the construction of mental simulation and generated positive product evaluation (Yim et al., 2018), this paper indicated that haptic cues in MVL, as an antecedent factor, could help consumers construct different types of mental simulations according to different types of products. This result fits consumers’ processing motives for products and could generate positive cognition and behavior. This is because different haptic cues are highly related to consumers’ motivation, and metaphors can narrow the conceptual tension among consumers, products, and haptic cues, thus increasing the familiarity and association among them.

### Managerial implications

First, facing a complex situation, marketers should have a comprehensive consideration of multi-factors, such as consumers’ psychological cognition, product type, medium characteristics, and many other factors. They need to choose from different visual tools to optimize communication effects. This paper designed haptic cues to change the consumers’ attitude toward online products with the help of visual language tools in order to compensate for the lack of tactile sensation of online consumers.

Second, this study is helpful for online retailers to pay attention to the role of consumer mental simulation on haptic salient attribute product perception, and select or adjust marketing strategies and means accordingly. Previously, when online retailers promoted products, most of them paid attention to whether the product price was reasonable. However, the important haptic attribute of the product itself was ignored by retailers, which was also one of the reasons for the high return rate of online products. It was found in this paper that consumers could produce different types of mental simulation for haptic salient attribute products, enlightening marketers that when the products sold are haptic salient attribute products, how to maximize consumers’ mental simulation of the products to enable consumers to achieve a shift in channel behavior (Li et al., 2021a,b) is the right marketing idea. For example, for tactile experiential products like blankets, hand haptic cues can enhance consumers’ mental simulation of the use of blankets, so consumers can perceive the softness and warmth of blankets in advance, and their favorable degree of products will be improved, so as to achieve better marketing effects.

Finally, this study also suggests that online retailers should seize the market segments of haptic salient attribute products and utilize haptic cues to improve the market acceptance of different products. In the past, when retailers carried out online sales, they often targeted large market segments and paid little attention to the market segments of haptic salient attribute products. As a result, they only grasped marketing data, but the corresponding marketing strategy and strategy principles were not clear. The results of this paper showed that different types of haptic cues should be set for products with different types of tactile salient attributes. Such as for blankets, pyjamas, and other tactile experiential products, consumers are more likely to imagine using their hands to touch the texture of products to make the evaluation. Therefore, it is more reasonable to set hand haptic cues for tactile experiential products. Similarly, for tactile functional products such as dumbbells, haptic object cues should be set to cater to haptic attributes such as weight, so as to improve shopping satisfaction.

### Limitations and future direction

First, although the valuation of products tended to increase when consumers touch products offline, it tended to decrease when others touch products through pollution effects (Argo et al., 2006). However, the pollution effect would lead to the weakening, blurring, and even distortion of consumers’ mental images in their minds, thus reducing the usefulness of their mental simulation (Baumgartner et al., 1992). Whether vicarious touch in an online environment also has pollution effects reducing the usefulness of mental simulations is a topic worth exploring. In addition to addressing the topic of adding haptic cues to achieve vicariously touch, Luangrath et al. (2022) showed that future studies could exaggerate the alternative touch effect of hand haptic cues (Schirmer and McGlone, 2019). In conjunction with the single object haptic cue used in this study, ould future research further exaggerate the vividness of object haptic cues as well? Could object haptic cues be designed as living rabbits to present the softness of pyjamas?

Second, the information collected by consumers could be attributed to the information presented in the stimulus (Miller, 1994), and the high load of product information description would also affect the mental simulation and, thus, affect the judgment (Chen and Lin, 2021). This paper used a haptic cue to explore their positive impact on consumers. When presented with information overload or haptic cues that were too complex to exceed the capacity of their transmission channels, this raises the possibility of target confusion (Gomes et al., 2020). Therefore, it is necessary for future research to study the load boundary of haptic cues, and further explore what type or quantity of haptic cues can play the most positive role under certain boundary conditions, while it will produce negative effects when exceeding this boundary.

Third, due to the limited sensory experience and expression skills, the experimental materials and design in this paper cannot fully meet the requirements of the expected research effect. In addition to viewing haptic cues, the generation of consumer mental simulation may also include the influence of other factors, such as touch devices used by consumers, virtual substitutes, auditory and haptic interaction, and other factors. Future research can further explore whether haptic cues and mental simulation also have positive effects based on interactive devices, or explore the influence of factors other than haptic attributes of products on consumers’ haptic perception.

Fourth, this paper mainly adopted the research method of simulated experiments with certain limitations. It can be considered to further carry out real field experiments to enhance the validity of external research.

## Data availability statement

The original contributions presented in this study are included in the article/supplementary material, further inquiries can be directed to the corresponding author.

## Author contributions

XL contributed to the empirical work, to the analysis of the results, and to the writing of the first draft. XZ, SW, and YX supported the total work of XL. XL contributed to overall quality and supervised the part of literature organization and empirical work. All authors discussed the results and commented on the manuscript.

## Appendix

**APPENDIX TABLE 1:** Scale.

Variables	Title items
Metaphorical control pre-test	Do you think this product display page contains two objects between the hand and the blanket, they are related (1 = strongly disagree, 7 = strongly agree)
	Do you think this product display page contains two objects between the hand and the blanket, they are similar (1 = strongly disagree, 7 = strongly agree)
	What do you think of the product’s detail display page? (1 = straightforward and responsive to facts, 7 = metaphorical and abstract)
Mental simulation pre-test (Xie et al., 2016)	When you are using this blanket, to what extent can you simulate the feeling of enjoying using this blanket? (e.g., touch it, feel the texture, weight, and softness) (1 = not at all, 7 = to a great extent)
	After you have used this blanket, to what extent can you simulate the feeling of enjoying using this blanket? (e.g., touch it, feel the texture, weight, and softness) (1 = not at all, 7 = to a great extent)
Process simulation (Xie et al., 2016)	When you viewed the blanket picture, how much did you think about the process of using and enjoying the blanket? (1 = not at all, 7 = very much)
	To what extent did images of using the blanket come to mind? (1 = not at all, 7 = to a great extent)
	Please indicate to what extent you could imagine using this blanket. (1 = not at all, 7 = to a great extent)
	How easy was it to imagine using this blanket? (1 = extremely difficult, 7 = extremely easy)
	How quickly did you start to think about using this blanket? (1 = not at all quickly, 7 = very quickly)
	How much do you agree or disagree with the statement that I had no difficulty imagining using and enjoying this blanket? (1 = strongly disagree, 7 = strongly agree)
Outcome simulation (Xie et al., 2016)	When you viewed the blanket picture, how much did you think about how you would feel after using the blanket? (1 = not at all, 7 = very much)
	To what extent did images of how you would feel after using the blanket come to mind? (1 = not at all, 7 = to a great extent)
	Please indicate to what extent you could imagine how you would feel after using the blanket. (1 = not at all, 7 = to a great extent)
	How easy was it to imagine how you would feel after using the blanket? (1 = extremely difficult, 7 = extremely easy)
	How quickly did you start to think about how you would feel after using the blanket? (1 = not at all quickly, 7 = very quickly)
	How much do you agree or disagree with the statement that I had no difficulty imagining how I would feel after using the blanket. (1 = strongly disagree, 7 = strongly agree)
Purchase intention (Bearden et al., 1984; Bone and Ellen, 1992; Biocca et al., 2001; Daugherty et al., 2008)	I will buy this one product (1 = “probably,” 7 = “very likely”)
	I will buy this one product (1 = “no probability,” 7 = “maximum probability”)
	I will buy this product (1 = “very uncertain,” 7 = “very certain”)
	I will buy this product (1 = “very unsure,” 7 = “very sure”)

Using the blanket as an example.
